# Semantic Communication for Intelligent Transmission and Recognition of High-Resolution Satellite Images in Satellite-to-Ground Systems

**DOI:** 10.3390/e28070803

**Published:** 2026-07-14

**Authors:** Jiaxin Liu, Qiwang Chen, Yijun Chen

**Affiliations:** Xiamen Key Laboratory of Mobile Multimedia Communications, College of Information Science and Engineering, Huaqiao University, Xiamen 361021, China

**Keywords:** semantic communication, very-high-resolution satellite imagery, remote sensing recognition tasks, vector quantization, semantic feature reweighting

## Abstract

Very-high-resolution (VHR) multispectral satellite imagery contains rich semantic information, yet its real-time transmission is constrained by limited satellite-to-ground bandwidth and dynamic channel impairments. Conventional communication schemes prioritize pixel-level reconstruction, resulting in large transmission overhead and poor robustness under unfavorable channel conditions. To address these challenges, an end-to-end task-oriented semantic communication framework for remote sensing downstream recognition tasks, termed Semantic Transmission Architecture for Remote Sensing (STARS), is proposed. To improve transmission efficiency for very-high-resolution remote sensing images with highly redundant background regions, a Semantic Feature Reweighting Module (SFRM) is introduced to dynamically evaluate token-level semantic importance and adaptively allocate transmission resources to task-critical features. Furthermore, vector quantization and a practical digital transmission chain are jointly integrated to achieve efficient semantic compression, while dynamic channel variations are incorporated during training to improve robustness under fading channel conditions. Experimental results on the DOTA dataset demonstrate that STARS consistently outperforms conventional schemes and existing semantic baselines under Rician fading channels, validating the effectiveness of semantic-aware feature allocation for bandwidth-efficient VHR imagery transmission.

## 1. Introduction

The evolution of 6G networks and the low-altitude economy imposes new demands on satellite-to-ground systems, particularly the need for massive connectivity and high-throughput data transmission. To meet these stringent demands and push the boundaries of spectral efficiency, significant advancements have been achieved in physical-layer designs. Specifically, innovative multiple access schemes, such as polarization-aided non-orthogonal multiple access (NOMA) [1], rate-diverse multiple access [2], and collision-resilient unsourced random access for massive MIMO [3], have been proposed to drastically improve channel capacity. Furthermore, advanced transceiver designs employing source-constrained hierarchical modulation with protograph LDPC codes have emerged as highly reliable solutions for IoT scenarios [4], while integrated sensing and communication (ISAC) architectures are maximizing spectrum utilization in modern sensor networks [5].

However, despite these remarkable physical-layer breakthroughs, the exponential growth in onboard Earth observation technologies has created a pronounced data gap with the severely limited downlink bandwidth of satellite–ground links.Very-high-resolution (VHR) and multispectral satellite imaging systems have entered the sub-meter era. The high spatial resolution, rich spectral information, and massive data volume of remote sensing imagery provide essential support for land resource monitoring, disaster response, and fine-grained urban management [6]. When applied to delay-sensitive tasks such as real-time object detection, the conventional “transmit everything first, interpret later on the ground” paradigm introduces substantial transmission latency and significantly constrains the intelligent responsiveness of Earth observation systems [7].

The fundamental bottleneck lies in the fact that traditional satellite image transmission strictly follows Shannon’s separated source and channel coding paradigm [8], which aims exclusively at bit-level error-free delivery. In this framework, images are treated as homogeneous bitstreams, completely ignoring the differences in semantic importance across the image content [9]. Under low-SNR or severely bandwidth-limited conditions, even conventional approaches with advanced physical-layer coding [4,10] suffer from the cliff effect and waste significant bandwidth on redundant background information. As a result, critical features related to fine-grained targets (e.g., ships, vehicles) are often irreparably distorted.

To break through the limitations of the traditional Shannon paradigm, semantic communication has emerged as a transformative paradigm for future 6G networks, laying a mathematical foundation for the semantic communications [11,12] and shifting the fundamental design philosophy from “how to accurately transmit bits” to “what meaning needs to be conveyed” [9,13]. By focusing on data significance and the specific goals of the receiver [14], task-oriented semantic communication extracts and transmits only the task-relevant features. Pioneering works in deep learning-enabled semantic communications (DeepSC) have demonstrated remarkable resilience against severe channel fading [15]. Particularly for vision-based and cross-modal applications, Deep Joint Source-Channel Coding (DeepJSCC) [16,17] and non-linear transform architectures [18] have been proposed. Moreover, task-oriented image transmission frameworks specifically designed for unmanned aerial systems [19] have shown the capability to efficiently extract and transmit critical features over wireless channels, effectively bypassing the traditional discrete quantization bottlenecks.

Despite these advances in generic image transmission, research on semantic communication systems tailored for very-high-resolution multispectral satellite imagery remains at an early stage. Most existing studies [15,16,18] focus on low-resolution natural images (e.g., CIFAR-10 or Kodak datasets) and emphasize visual reconstruction, without adequately considering the inherent complexities of remote sensing imagery. Specifically, three unique challenges arise for VHR satellite images:

1. Extreme large-background-small-target distribution:Task-critical objects (e.g., ships, vehicles, aircraft) occupy only a tiny fraction of pixels, while the vast majority are semantically redundant backgrounds. Directly applying generic image JSCC would waste limited bandwidth on transmitting irrelevant features.
2. Wide object scale variation and complex textures: Objects in satellite imagery span an extremely wide scale range (from small vehicles to large harbors), requiring hierarchical multi-scale representations that standard CNNs or Vision Transformers (ViTs) cannot efficiently capture without prohibitive computational cost.
3. Strict onboard resource constraints: Satellite payloads have severely limited power, memory, and computing capabilities, demanding lightweight yet powerful feature extraction.

Most related to our work are several recent efforts that also explore task-oriented discrete semantic transmission. Chen et al. [20] proposed VQ-DSC-R, a vector quantized-enabled digital semantic communication system built upon OFDM. Similar to our approach, VQ-DSC-R adopts a Swin Transformer backbone and utilizes an exponential moving average (EMA) mechanism for codebook updates. However, VQ-DSC-R focuses on compression and reconstruction quality under OFDM, whereas our proposed framework explicitly optimizes for downstream detection accuracy and employs a full digital chain (LDPC and 16-QAM) compatible with existing satellite ground infrastructure. For remote sensing images, the ASE-JSCC framework [21] introduced a semantic extraction module coupled with a feature selection mechanism, achieving 84.29–88.62% classification accuracy at a compression ratio of 384:1. While ASE-JSCC demonstrates the feasibility of task-oriented compression for remote sensing, its feature selection is not adaptive to instantaneous channel conditions. Bui et al. [22] proposed a semantic encoding strategy for change detection in multispectral satellite images, where pixel importance scores are computed to guide transmission. This aligns with our philosophy of prioritizing task-relevant content, while their importance evaluation remains static and fixed regardless of the input scene, lacking the ability to adapt to varying semantic densities across different VHR images. Collectively, these studies validate the feasibility of task-oriented semantic communication for remote sensing applications. However, these approaches primarily focus on pixel-level image reconstruction rather than intelligent semantic recognition. Furthermore, existing works still lack a unified framework that jointly integrates practical digital transmission mechanisms, adaptive semantic prioritization for highly imbalanced scene content, and cross-layer end-to-end optimization under dynamic fading channels for downstream intelligent recognition.

The main contributions of this paper are summarized as follows:

- A task-oriented digital semantic transmission framework for satellite–ground communications: To overcome the limitations of conventional semantic communication systems that directly transmit latent features over simplified analog channels, a unified task-oriented semantic transmission framework is developed for satellite–ground remote sensing applications. By incorporating vector quantization together with practical digital transmission components, including channel coding, modulation, fading channels, demodulation, and channel decoding, the proposed framework enables end-to-end semantic transmission under realistic wireless communication conditions.
- End-to-end optimization under dynamic wireless channel conditions: To improve transmission reliability in time-varying satellite communication environments, our framework jointly optimizes semantic feature extraction, discrete semantic quantization, and physical-layer transmission distortions in an end-to-end manner. By explicitly incorporating dynamic channel variations into the training process, the framework learns robust semantic representations that maintain stable downstream task performance under severe fading conditions.
- Adaptive Semantic Feature Reweighting Module (SFRM): To overcome the limitation of existing semantic masking strategies that assign a uniform compression ratio to all transmitted features, a novel Semantic Feature Reweighting Module (SFRM) is introduced for adaptive semantic transmission. Instead of treating all semantic features equally during transmission, SFRM performs content-aware feature evaluation and dynamically allocates different masking ratios according to the task relevance of each semantic token. As a result, critical target-related features retain more semantic information, whereas redundant background regions are compressed more aggressively, significantly improving transmission efficiency under bandwidth-limited conditions.

The rest of this article unfolds as follows. Section 2 lays the groundwork by introducing the end-to-end task-oriented architecture and the corresponding channel model. The technical core of STARS is detailed in Section 3, which dissects the Swin Transformer backbone, the proposed SFRM, and the vector quantization process. Section 4 subsequently tackles the end-to-end training challenges via a multi-task loss formulation and an SNR curriculum learning scheme. Extensive experimental validations and ablation studies under diverse channel environments are discussed in Section 5, before concluding remarks and future outlooks are offered in Section 6.

## 2. System Overview

We propose STARS (Semantic Transmission Architecture for Remote Sensing), an end-to-end task-oriented semantic communication framework designed for efficient satellite-to-ground transmission of very-high-resolution remote sensing imagery. Unlike conventional communication systems that focus on accurate pixel-level reconstruction, STARS directly optimizes the transmission process toward downstream semantic understanding tasks, enabling more efficient utilization of constrained satellite communication resources.

**Figure 1:**
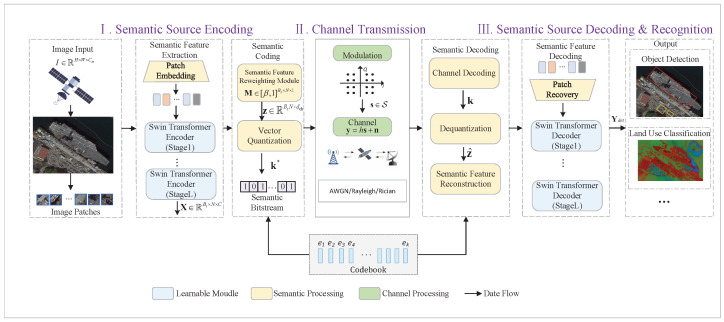
Semantic Transmission Architecture for Remote Sensing.

As illustrated in Figure 1, the overall architecture of STARS consists of three sequential processing stages: onboard semantic transmitter processing, wireless channel transmission, and ground-based semantic receiver processing. At the transmitter side, the input remote sensing image is first processed by a hierarchical semantic encoder to extract multi-scale feature representations. A Semantic Feature Reweighting Module (SFRM) then dynamically emphasizes task-relevant regions while suppressing redundant background information. The refined semantic features are subsequently compressed into discrete semantic codewords through vector quantization and converted into digital bitstreams for physical-layer transmission. After propagating through the wireless channel, the received signal is demodulated and channel-decoded at the receiver side to recover the transmitted semantic information. Finally, the reconstructed semantic features are fed into the semantic decoder, which directly performs downstream intelligent tasks such as remote sensing object detection.

### 2.1. Semantic Transmitter Processing

The semantic transmitter is responsible for extracting task-relevant information from the input remote sensing image and converting it into compact semantic representations suitable for wireless transmission. As illustrated in Figure 1, the transmitter performs three sequential operations: hierarchical semantic feature extraction, semantic feature reweighting, and discrete semantic quantization.

First, the input VHR multispectral image, denoted as $I\in R^{H\times W\times C_{in}}$, is processed by a hierarchical Swin Transformer backbone [23]. Instead of outputting a generic global vector, this backbone extracts multi-scale continuous semantic features, formulated as a tensor $X\in R^{B_{s}\times N\times C}$ (the specific topological advantages of which are detailed in Section 3.1).

To further suppress redundant background information, the extracted features are passed through a Semantic Feature Reweighting Module (SFRM). Acting as a content-aware attention gate, the SFRM generates a dynamic mask $M\in {\left[\beta ,1\right]}^{B_{s}\times N\times 1}$ and performs element-wise semantic reweighting to yield the refined feature tensor $\hat{X}=X⊙M$, where ⊙ denotes the element-wise Hadamard product. This operation explicitly suppresses task-irrelevant background tokens while preserving critical semantics, ensuring that only high-density information proceeds to the physical layer.

Subsequently, the refined feature tensor $\hat{X}$ is linearly projected to a lower-dimensional space to match the channel capacity, yielding the continuous intermediate feature $z\in R^{B_{s}N\times d_{\mathrm{ch}}}$. To seamlessly integrate this semantic representation with modern digital space–ground networks, a vector quantization (VQ) mechanism is employed. By utilizing a shared discrete semantic codebook $E\in R^{K\times d_{\mathrm{ch}}}$, where $K=2^{B}$ defines the total quantization capacity governed by the allocation bit-width *B*, the quantizer maps each continuous feature vector to its nearest codeword, outputting the discretized semantic features $z_{q}$ alongside a highly compact 1D index sequence $k^{*}$.

Finally, the index sequence is converted into an information bit sequence b, which is subsequently encoded by an LDPC encoder to generate the coded bit sequence c. The coded bits are then mapped to complex modulation symbols s using 16-QAM. Let S denote the standard 16-QAM constellation set. The transmitted symbols satisfy the average power constraint

(1) $$\frac{1}{N_{s}}\sum_{i=1}^{N_{s}}{\left|s_{i}\right|}^{2}\leq P,$$

where *P* denotes the average power per transmitted symbol, $s_{i}\in S$ denotes the *i*-th transmitted complex symbol, and $N_{s}$ is the total number of transmitted symbols.

### 2.2. Channel Model and Receiver Processing

During the satellite-to-ground downlink, the modulated complex symbol vector s inevitably experiences channel fading and additive noise. In this work, the satellite channel is modeled as a Rician fading channel to provide a generalized formulation for space–ground wireless propagation. This model is widely adopted because satellite links typically maintain a dominant line-of-sight (LoS) component, while the high relative mobility of satellites causes the propagation environment to be highly dynamic and time-varying [24]. Assuming a flat block fading model where the channel state remains constant over a transmission block, the received signal vector is expressed as y=hs+n, where $n∼\mathrm{CN}(0,\;\sigma^{2}I_{N_{s}})$ represents the complex Additive White Gaussian Noise (AWGN) vector, with $I_{N_{s}}$ being the identity matrix. The complex Rician fading coefficient *h* is defined as

(2) $$h=\sqrt{\frac{K_{R}}{K_{R}+1}}h_{\mathrm{LoS}}+\sqrt{\frac{1}{K_{R}+1}}h_{\mathrm{NLoS}},$$

where $K_{R}$ denotes the Rician *K*-factor, representing the power ratio between the deterministic LoS component and the diffuse NLoS components. The term $h_{\mathrm{LoS}}$ represents the deterministic LoS component with $|h_{\mathrm{LoS}}|=1$, and $h_{\mathrm{NLoS}}∼\mathrm{CN}\left(0,1\right)$ denotes the scattering NLoS component. Particularly, when $K_{R}=0$, the Rician fading channel degenerates into the conventional Rayleigh fading channel. Unless otherwise specified, all simulations in this work are conducted under the setting $K_{R}=0.1$. Accordingly, the instantaneous SNR of this link is expressed as $\gamma =\frac{{P|h|}^{2}}{\sigma^{2}}$, where *P* is the average symbol power defined above.

To maximize the decoding performance under fading conditions, the ground receiver employs a soft-decision demapping strategy. For the *k*-th received scalar symbol $y_{k}$, it computes the log-likelihood ratios (LLRs) for each mapped coded bit $c_{j}$ by iterating over the constellation set S:

(3) $$\mathrm{LLR}\left(c_{j}\right)=\log\frac{\sum_{s^{\prime }\in S:c_{j}=0}\exp\left(-\frac{|y_{k}-h\cdot s^{\prime }{|}^{2}}{\sigma^{2}}\right)}{\sum_{s^{\prime }\in S:c_{j}=1}\exp\left(-\frac{|y_{k}-h\cdot s^{\prime }{|}^{2}}{\sigma^{2}}\right)},$$

where $s^{\prime }\in S$ represents the trial constellation point during the soft demapping search. These LLRs serve as the soft-information interface, which is fed into the LDPC decoder using the Min-Sum Belief Propagation algorithm to correct channel-induced bit flips and accurately recover the estimated index sequence $\hat{k}$.

By performing a symmetric lookup operation in the shared codebook E, the ground station reconstructs the dequantized estimated semantic feature tensor $\hat{z}$. Finally, without the need to explicitly reconstruct the raw pixel-level image, these recovered semantic features are processed by a hierarchical task decoder to directly output the object detection predictions ${\hat{Y}}_{det}$, successfully completing the end-to-end task-oriented transmission loop.

## 3. Semantic Component Design and Mathematical Formulation

To achieve efficient data downloading under stringent physical constraints while providing a robust feature basis for downstream object detection tasks, this section details the internal mathematical formulation and algorithmic design of the core components in the STARS framework.

### 3.1. Hierarchical Semantic Backbone with Swin Transformer

Semantic communication shifts the design goal from accurate bit transmission to the effective delivery of task-relevant meaning. For VHR satellite images, this requirement primarily confronts two major challenges: wide object scale variation and complex textures and strict onboard resource constraints. Standard Vision Transformers (ViTs) offer global self-attention but suffer from a quadratic computational complexity that scales with the total number of image pixels, making them computationally prohibitive for deployment on resource-constrained satellite nodes.

To overcome these limitations, we adopt the Swin Transformer as the core feature extraction backbone. By restricting the computation of self-attention to non-overlapping local windows, the Swin Transformer significantly reduces the computational overhead to linear complexity relative to the image size, thereby accommodating stringent onboard computational limits.

**Figure 2:**
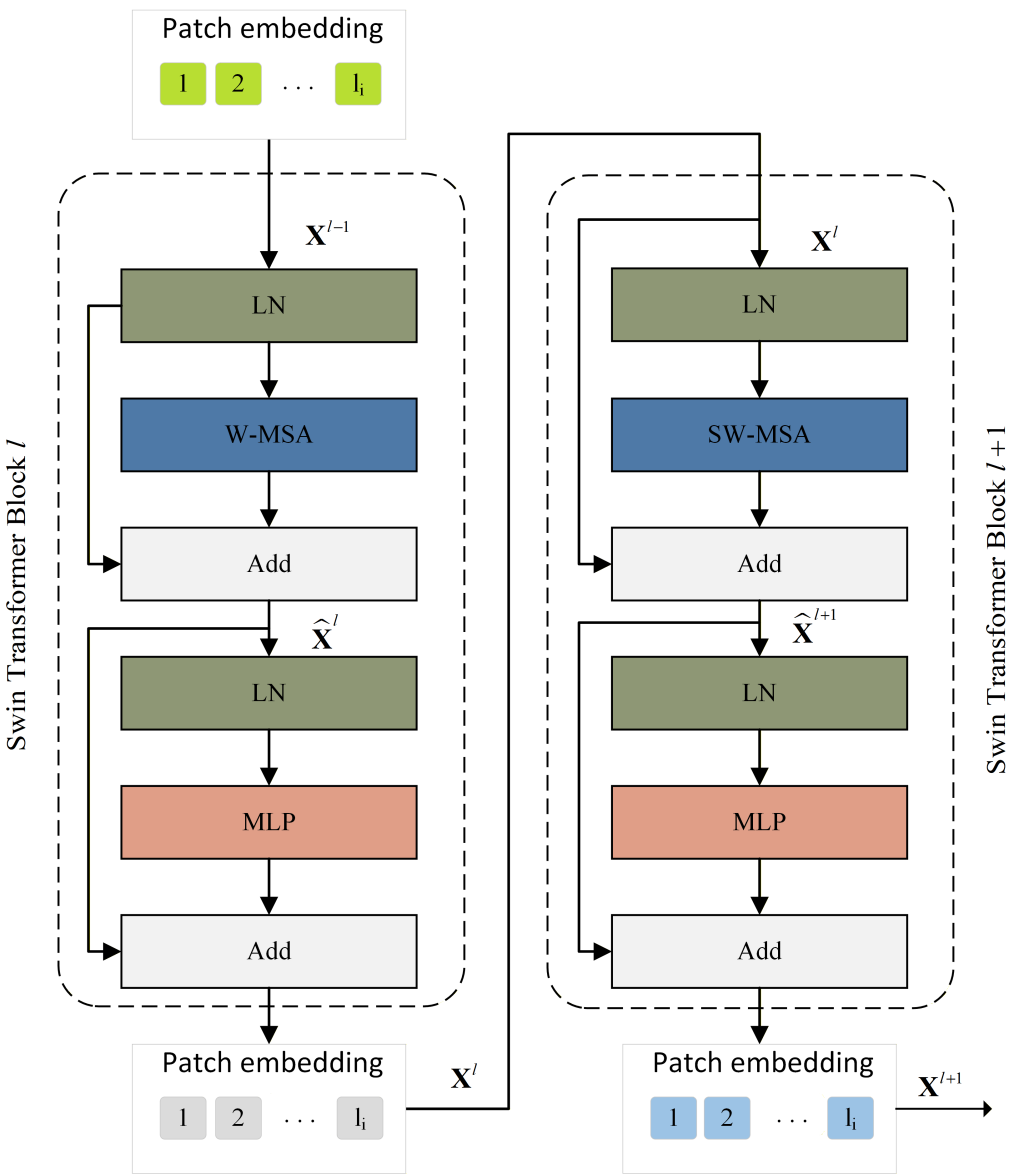
Architecture of the Swin Transformer-based semantic backbone with shifted window mechanism.

Mathematically, given the feature map $X^{l-1}$ at the (l−1)-th layer, the continuous hierarchical representation is updated by alternately applying the Window Multi-head Self-Attention (W-MSA) and Shifted Window Multi-head Self-Attention (SW-MSA) across consecutive blocks, as illustrated in Figure 2:

(4) $$\;{\hat{X}}^{l}=\text{W-MSA}\left(\mathrm{LN}\left(X^{l-1}\right)\right)+X^{l-1},$$

(5) $$X^{l}=\mathrm{MLP}\left(\mathrm{LN}\left({\hat{X}}^{l}\right)\right)+{\hat{X}}^{l},$$

(6) $$\;{\hat{X}}^{l+1}=\text{SW-MSA}\left(\mathrm{LN}\left(X^{l}\right)\right)+X^{l},$$

(7) $$\;X^{l+1}=\mathrm{MLP}\left(\mathrm{LN}\left({\hat{X}}^{l+1}\right)\right)+{\hat{X}}^{l+1},$$

where LN(·) represents Layer Normalization, and the MLP consists of two fully connected layers coupled with a GELU activation.

While Equations (4)–(7) describe the macroscopic layer updates, both W-MSA and SW-MSA rely on the same underlying self-attention mechanism. To clarify how they operate, we first define their spatial partitioning strategies. W-MSA computes self-attention only within isolated, non-overlapping local windows of size M×M. Although this design successfully bounds the computational complexity, it completely cuts off information exchange between adjacent windows. To resolve this, SW-MSA is applied in the subsequent layer. It shifts the window partitioning grid by (⌊M/2⌋,⌊M/2⌋) patches, effectively bridging the boundaries of the preceding windows and enabling cross-window feature interactions.

Inside any given local window—whether standard or shifted—the spatial tokens are linearly projected into query (Q), key (K), and value (V) matrices. The internal attention weights are then calculated as follows:

(8) $$\mathrm{Attention}\left(Q,K,V\right)=\mathrm{SoftMax}\left(\frac{QK^{T}}{\sqrt{d_{q}}}+B_{\mathrm{pos}}\right)V,$$

where $Q,K,V\in R^{M^{2}\times d_{q}}$, and $d_{q}$ is the query/key dimension. Notice that we do not rely solely on the dot product of queries and keys. Because remote sensing objects vary significantly in scale and orientation, we explicitly add a relative position bias $B_{\mathrm{pos}}\in R^{M^{2}\times M^{2}}$ to encode their spatial topologies. This specific formulation guarantees that the resulting continuous semantic tensor $X\in R^{B_{s}\times N\times C}$ maintains spatial translation invariance, which provides a robust feature foundation for the downstream detection task.

### 3.2. Semantic Feature Reweighting Module for Content-Aware Masking

VHR remote sensing images often contain highly redundant background information, while critical target objects are sparsely distributed across the scene. Although the Swin Transformer backbone captures hierarchical semantic representations effectively, the dense feature tensor X still encapsulates a considerable amount of background-induced responses. To allocate constrained transmission bandwidth exclusively to critical targets, we propose a Semantic Feature Reweighting Module (SFRM) operating directly in the high-dimensional latent space.

**Figure 3:**
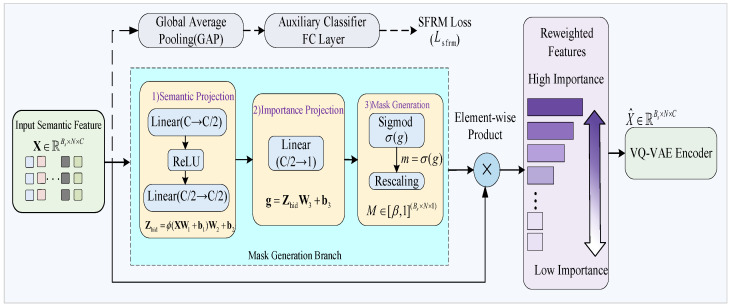
Architecture of the Semantic Feature Reweighting Module (SFRM).

As illustrated in Figure 3, the SFRM functions as a content-aware non-linear gating mechanism to filter out background features. The core of this module includes a Mask Generation Branch, which dynamically evaluates the token-level importance to suppress redundant information. For each spatial token in the continuous feature X, the module extracts a customized task-relevant score. Formally, the token representations are first projected into a hidden semantic space through a dual-layer perceptron with ReLU activation ϕ(·):

(9) $$Z_{\mathrm{hid}}=\phi \left(XW_{1}+b_{1}\right)W_{2}+b_{2},$$

where $W_{1},W_{2}$ and $b_{1},b_{2}$ are learnable weight matrices and biases. Subsequently, a linear gating layer maps $Z_{\mathrm{hid}}$ to a logit tensor g, followed by a Sigmoid function to generate the raw normalized importance score $m\in {\left[0,1\right]}^{B_{s}\times N\times 1}$:

(10) $$g=Z_{\mathrm{hid}}W_{3}+b_{3},$$

(11) m=Sigmoid(g).

Directly utilizing m as a binary or near-zero mask would aggressively eliminate background tokens, leading to catastrophic gradient vanishing during backpropagation and destroying the macroscopic contextual clues. Therefore, we introduce a bounded rescaling transformation to derive the final dynamic mask M:

(12) M=β+(1−β)·m,

where β∈(0,1) acts as a protective lower bound. Empirically, we set β=0.3 based on a structural trade-off: a lower bound below 0.2 induces severe gradient vanishing for background tokens during training, while a bound above 0.5 fails to sufficiently suppress semantically redundant information. The chosen value of 0.3 optimally maintains the essential gradient flow while achieving approximately a 70% mathematical attenuation of background features. The final element-wise reweighting is executed as $\hat{X}=X⊙M$. This mathematical design ensures that target tokens are exponentially amplified for high-fidelity quantization, while background tokens are adaptively suppressed without completely severing the gradient flow. The complete forward behavior of the SFRM is summarized in Algorithm 1.
**Algorithm 1** Forward Process of the Content-Aware SFRM**Input:** Dense continuous patch features $X\in R^{B_{s}\times N\times C}$
   1:// *Local feature refinement via self-attention block*   2:$\tilde{X}\leftarrow \mathrm{LayerNorm}\left(X\right)$   3:$X\leftarrow X+\mathrm{DropPath}(\mathrm{MSA}\left(\tilde{X}\right))$   4:X←X+DropPath(MLP(LayerNorm(X)))   5:X←ReLU(X)   6:// *Auxiliary downstream recognition for semantic guidance*   7:$x_{\mathrm{pool}}\leftarrow \mathrm{MeanPool}\left(X,\dim=C\right)$   8:$p_{\mathrm{aux}}\leftarrow \mathrm{FC}\left(x_{\mathrm{pool}}\right)$   9:// *Gating network for dynamic mask generation* 10:$Z_{\mathrm{hid}}\leftarrow \mathrm{ReLU}\left(XW_{1}+b_{1}\right)W_{2}+b_{2}$ 11:$g\leftarrow Z_{\mathrm{hid}}W_{3}+b_{3}$ 12:m←Sigmoid(g) 13:// *Bounded rescaling and element-wise masking* 14:M←β+(1−β)·m 15:$\hat{X}\leftarrow X⊙M$
**Output:** Reweighted semantic features $\hat{X}$, auxiliary task logits $p_{\mathrm{aux}}$


### 3.3. Semantic Codebook and Vector Quantization

To deliver robust feature representations over degraded satellite downlink channels while adhering to stringent onboard computational limits, the continuous reweighted features $\hat{X}$ must be mapped onto a discrete semantic space. First, a linear projection matrix $W_{\mathrm{enc}}\in R^{d_{\mathrm{ch}}\times C}$ and a bias vector $b_{\mathrm{enc}}\in R^{d_{\mathrm{ch}}}$ are introduced to compress the continuous semantic channel dimension:

(13) $$z_{i}={\left(\hat{X}\right)}_{i}W_{\mathrm{enc}}^{T}+b_{\mathrm{enc}},\;\forall i\in \left\{1,\ldots ,B_{s}\times N\right\},$$

yielding the pre-quantization continuous latent feature vector $z_{i}\in R^{d_{\mathrm{ch}}}$, where $d_{\mathrm{ch}}$ is the reduced channel dimension.

We establish a discrete semantic codebook $E=\left\{e_{1},e_{2},\ldots ,e_{K}\right\}\in R^{K\times d_{\mathrm{ch}}}$, which is shared symmetrically between the transmitter and the receiver. Here $K=2^{B}$ defines the total quantization capacity, with *B* denoting the number of bits allocated per latent feature dimension. The VQ process performs a non-linear Voronoi partition of the continuous manifold. For each incoming continuous vector $z_{i}$, the quantizer executes a nearest-neighbor Euclidean distance matching across the codebook space to output the optimal codeword index $k_{i}^{*}$:

(14) $$k_{i}^{*}=\arg\min_{k\in \{1,\ldots ,K\}}{∥z_{i}-e_{k}∥}_{2}^{2}.$$

Consequently, the continuous vector $z_{i}$ is snapped to its closest discrete basis vector, yielding the quantized representation $z_{q,i}=e_{k_{i}^{*}}$.

#### Codebook Optimization via EMA and Dead Codeword Reset

During the end-to-end training phase, updating the discrete codebook E via standard gradient descent often induces severe gradient variance. Specifically, because the non-differentiable argmin operation restricts standard gradient backpropagation, direct optimization of the discrete codebook via the task loss is infeasible. To guarantee smooth convergence of the semantic clusters under stringent onboard computational limits, we employ an Exponential Moving Average (EMA) mechanism [25] to dynamically update the codewords. The EMA mechanism serves as an online, gradient-free clustering approach. It smoothly updates each codeword toward the moving average of the continuous feature vectors assigned to it, which guarantees training stability and prevents codebook collapse. Let I(·) denote the indicator function. The instantaneous activation frequency $f_{k}^{\left(t\right)}$ of the *k*-th codeword within the current training batch at iteration *t* is formulated as

(15) $$f_{k}^{\left(t\right)}=\sum_{i=1}^{B_{s}\times N}I\left(k_{i}^{*}=k\right).$$

To track the long-term usage history and directional semantic trajectories of the latent features, the accumulated cluster volume $V_{k}^{\left(t\right)}$ and the continuous feature embedding sum $v_{k}^{\left(t\right)}$ are updated via a smoothing decay rate $\alpha_{\mathrm{ema}}\in \left(0,1\right)$:

(16) $$\;V_{k}^{\left(t\right)}=\alpha_{\mathrm{ema}}V_{k}^{\left(t-1\right)}+\left(1-\alpha_{\mathrm{ema}}\right)f_{k}^{\left(t\right)},$$

(17) $$v_{k}^{\left(t\right)}=\alpha_{\mathrm{ema}}v_{k}^{\left(t-1\right)}+\left(1-\alpha_{\mathrm{ema}}\right)\sum_{i=1}^{B_{s}\times N}z_{i}\cdot I\left(k_{i}^{*}=k\right).$$

Then, the updated codeword $e_{k}^{\left(t\right)}$ is derived by executing a structural normalization $e_{k}^{\left(t\right)}=v_{k}^{\left(t\right)}/V_{k}^{\left(t\right)}$.

Furthermore, a critical pathological vulnerability in task-oriented remote sensing transmission is the presence of a severe long-tail target distribution, where uninformative backgrounds dominate the latent space. Under such conditions, standard EMA tracking suffers from the codebook collapse phenomenon, where a small subset of active codewords is frequently updated, while the remaining vast majority of codewords are never matched, rendering them barren placeholders or dead codes. To preserve the maximum representative capacity of the discrete semantic space, we integrate an active Dead Codeword Reset Mechanism directly into the update loop. We explicitly define a structural utilization threshold $\epsilon =10^{-4}$. At the end of each training epoch, the system monitors the accumulated cluster volume $V_{k}^{\left(t\right)}$ for all k∈{1,…,K}. If a codeword’s tracking value falls below the threshold, it is mathematically diagnosed as a dead code and reinitialized as

(18) $$e_{k}^{\left(t\right)}\leftarrow z_{\mathrm{rand}}\;\mathrm{if}\;V_{k}^{\left(t\right)}<\epsilon ,$$

where $z_{\mathrm{rand}}$ is a high-density continuous feature vector randomly sampled from the current training batch. This reinitialization forcibly revives underutilized dead slots into active live codes, ensuring that the entire codebook capacity is globally optimized to compress and deliver intricate geospatial details under extreme bandwidth constraints. The complete discretization and codebook optimization workflow is formulated in Algorithm 2.
**Algorithm 2** Discrete Vector Quantization and Codebook Optimization**Input:** Compressed features $z\in R^{B_{s}N\times d_{\mathrm{ch}}}$, codebook $E\in R^{K\times d_{\mathrm{ch}}}$
   1:Compute pairwise $L_{2}$ distances: $d\left(z_{i},e_{k}\right)=\left|\right|z_{i}{\left|\right|}^{2}+\left|\right|e_{k}{\left|\right|}^{2}-2z_{i}^{⊤}e_{k}$   2:Find nearest codebook indices via Voronoi matching: $k_{i}^{*}\leftarrow \arg\min_{k}d\left(z_{i},e_{k}\right)$   3:Quantize features to closest basis vector: $z_{q,i}\leftarrow E\left[k_{i}^{*}\right]$   4:Compute VQ commitment loss: $L_{\mathrm{commit}}\leftarrow \frac{1}{B_{s}N}\sum_{i}||\mathrm{sg}\left[z_{q,i}\right]-z_{i}{\left|\right|}^{2}$   5:Compute VQ codebook loss: $L_{\mathrm{embed}}\leftarrow \frac{1}{B_{s}N}\sum_{i}\left|\right|z_{q,i}-\mathrm{sg}\left[z_{i}\right]{\left|\right|}^{2}$   6:Total vector quantization loss formulation: $L_{\mathrm{VQ}}\leftarrow \eta L_{\mathrm{commit}}+L_{\mathrm{embed}}$   7:**if** training mode **then**   8:    Update cluster counts: $V_{k}^{\left(t\right)}\leftarrow \alpha_{\mathrm{ema}}V_{k}^{\left(t-1\right)}+\left(1-\alpha_{\mathrm{ema}}\right)\mathrm{count}\left(k\right)$   9:    Update embedding sums: $v_{k}^{\left(t\right)}\leftarrow \alpha_{\mathrm{ema}}v_{k}^{\left(t-1\right)}+\left(1-\alpha_{\mathrm{ema}}\right)\sum_{k_{i}^{*}=k}z_{i}$ 10:    **Corrected** Laplace smoothing: ${\tilde{V}}_{k}^{\left(t\right)}\leftarrow \frac{V_{k}^{\left(t\right)}+\epsilon_{0}}{\sum_{j}V_{j}^{\left(t\right)}+K\epsilon_{0}}\sum_{j}V_{j}^{\left(t\right)}$ 11:    Update active codewords via structural normalization: $e_{k}^{\left(t\right)}\leftarrow v_{k}^{\left(t\right)}/{\tilde{V}}_{k}^{\left(t\right)}$ 12:    **if** $V_{k}^{\left(t\right)}<10^{-4}$ **then** 13:        Reset dead code slots to active features: $e_{k}^{\left(t\right)}\leftarrow z_{\mathrm{rand}}$ 14:    **end if** 15:**end if** 16:Straight-through estimator (STE) gradient approximation: $z_{q}\leftarrow z+\mathrm{sg}\left[z_{q}-z\right]$
**Output:** Quantized features $z_{q}$, vector quantization loss $L_{\mathrm{VQ}}$


## 4. End-to-End Joint Optimization and Training Strategy

To achieve end-to-end optimization across a non-differentiable physical channel, we propose a channel-in-the-loop training paradigm. This framework systematically integrates a Straight-Through Estimator (STE) for gradient approximation, a multi-task loss formulation for semantic fidelity, and a curriculum learning scheduler to ensure convergence stability under complex remote sensing communication environments.

### 4.1. Channel-in-the-Loop Paradigm and Straight-Through Estimator

Because vector quantization, LDPC encoding/decoding, and QAM modulation fundamentally involve non-differentiable discrete operations, the entire physical transmission layer blocks standard gradient backpropagation. To address this mathematical bottleneck, we employ a Straight-Through Estimator (STE).

During the forward pass, the data strictly executes the complete physical chain (quantization → modulation → fading channel → demodulation). However, during backpropagation, the gradient mathematically bypasses the physical layer and flows directly from the received dequantized feature $\hat{z}$ back to the sender’s continuous feature z:

(19) $$z_{q}=z+\mathrm{sg}\left[\hat{z}-z\right],$$

where sg[·] denotes the stop-gradient operator, which behaves as an identity mapping during the forward pass while blocking gradient propagation during backpropagation. This formulation ensures that the onboard Swin encoder receives meaningful task-oriented gradients while the non-differentiable physical layer is gracefully bypassed.

### 4.2. Joint Optimization Objectives

To explicitly enforce the fidelity of semantic features against physical channel impairments, we formulate a multi-task loss function. The total end-to-end objective is defined as

(20) $$L_{\mathrm{total}}=L_{\mathrm{cls}}+\lambda_{\mathrm{sfrm}}L_{\mathrm{sfrm}}+\lambda_{\mathrm{vq}}L_{\mathrm{vq}}+\lambda_{\mathrm{fid}}L_{\mathrm{fid}},$$

where the individual components are carefully balanced by hyperparameter weights ($\lambda_{\mathrm{sfrm}},\lambda_{\mathrm{vq}},\lambda_{\mathrm{fid}}$), and defined as follows:

- $L_{\mathrm{cls}}$: The primary cross-entropy classification loss driving the downstream recognition task.
- $L_{\mathrm{sfrm}}$: The auxiliary cross-entropy loss guiding the gating network within the SFRM module.
- $L_{\mathrm{vq}}$: The vector quantization loss, comprising the commitment loss and the codebook loss to align the continuous manifolds with the discrete basis vectors:
  (21) $$L_{\mathrm{vq}}={∥z_{q}-\mathrm{sg}\left[z\right]∥}_{2}^{2}+\eta {∥\mathrm{sg}\left[z_{q}\right]-z∥}_{2}^{2},$$
  where η is the commitment cost coefficient.
- $L_{\mathrm{fid}}$: The semantic fidelity loss directly computing the Mean Squared Error (MSE) between the received feature and the transmitted continuous feature:
  (22) $$L_{\mathrm{fid}}={∥\hat{z}-\mathrm{sg}\left[z\right]∥}_{2}^{2}.$$

By allowing gradients to flow through $\hat{z}$ in $L_{\mathrm{fid}}$, the encoder receives direct penalization feedback regarding channel-induced distortions, forcing it to learn representations that are inherently robust to fading.

### 4.3. SNR Curriculum Learning Strategy

A critical challenge in training the channel-in-the-loop framework is the gradient divergence caused by severe channel noise during the initial training stages. If the network is exposed to extreme fading channels (e.g., −12 dB) from the very first epoch, the completely untrained Swin encoder fails to extract meaningful semantic clusters, and the backpropagated gradients from the semantic fidelity loss become overwhelmingly noisy and destructive.

To address this, we design a SNR curriculum learning strategy. The training process adopts an easy-to-hard progressive learning scheme. In the early training phase, the physical channel SNR is uniformly sampled from a benign, high-SNR range. This optimal environment allows the Swin encoder and the discrete codebook to rapidly establish a clean, highly discriminative semantic latent space.

As training proceeds and the semantic clusters stabilize, the lower bound of the SNR sampling distribution is gradually decayed. The system is thereby exposed to progressively harsher fading conditions until the full challenging target range is covered. This curriculum strategy not only prevents early-stage codebook collapse but also significantly improves final convergence stability, ensuring exceptional robustness under extreme noise levels in Rician fading channels.

To validate the proposed SNR curriculum learning strategy, we compared the convergence behavior with and without curriculum learning. As shown in Table 1, the proposed strategy converges earlier and reaches early stopping sooner, demonstrating improved training efficiency and convergence stability.

## 5. Simulation Results and Analysis

In this section, we conduct extensive experiments to evaluate the performance of the proposed STARS framework. We first introduce the dataset and implementation details, followed by an analysis of the system performance under various channel conditions and ablation studies.

### 5.1. Dataset and Preprocessing

To evaluate the performance of the proposed robust semantic communication system in high-resolution satellite image transmission and downstream recognition tasks, we employ the large-scale aerial image dataset DOTA-v1.0 [1]. The imagery in DOTA exhibits significant diversity, comprising data collected from Google Earth, the Gaofen-2 (GF-2) and Jilin-1 (JL-1) satellites provided by the China Centre for Resources Satellite Data and Application, and aerial images from CycloMedia BV. Depending on the sensor payloads, the dataset includes both multispectral RGB images and grayscale images generated from the panchromatic bands of GF-2 and JL-1. This multi-source and heterogeneous data composition effectively simulates the complex image attributes encountered in low-altitude economy and Earth observation scenarios, thereby validating the generalization capability of our semantic encoder across different imaging modalities.

For object categories, we utilize the DOTA-v1.0 annotations, which encompass 15 common geospatial object categories: Plane, Ship, Storage Tank, Baseball Diamond, Tennis Court, Basketball Court, Ground Track Field, Harbor, Bridge, Large Vehicle, Small Vehicle, Helicopter, Roundabout, Soccer Ball Field, and Swimming Pool. Given the extreme variations in object scales and arbitrary orientations within aerial scenes, the annotations are provided in the form of Oriented Bounding Boxes (OBBs).

### 5.2. Experimental Setup and Implementation Details

The detailed architectural configuration and the intermediate feature dimensions for each module of the proposed STARS framework are summarized in Table 2. The proposed STARS system is implemented using the PyTorch 2.5.1 deep learning framework on an Ubuntu 22.04 operating system. All models are trained and evaluated on a workstation equipped with an Intel Core i9 CPU and four NVIDIA RTX 4090 GPUs.

Evaluation Metrics: To comprehensively evaluate the proposed STARS framework, we adopt a primary-auxiliary metric strategy to assess both semantic transmission reliability and downstream task utility:

- Classification Accuracy: This metric measures the intrinsic transmission robustness of the semantic system, evaluating how well categorical semantic information is preserved under varying SNRs.
- mAP@0.5: To evaluate the fine-grained spatial localization for downstream object detection, this metric is calculated at a fixed IoU threshold of 0.5, where a detection is considered correct if the predicted bounding box overlaps with the ground-truth box by at least 50%. It serves as a standard baseline for assessing coarse localization performance.
- mAP@0.5:0.95: This metric averages the mAP over ten IoU thresholds from 0.5 to 0.95 with a step size of 0.05. It provides a more stringent evaluation by increasingly penalizing imprecise bounding boxes at higher IoU levels.

### 5.3. Results Analysis

#### 5.3.1. Effectiveness of the Semantic Feature Reweighting Module

To evaluate the effectiveness of the proposed SFRM, ablation experiments were conducted on the DOTA dataset using 1024×1024 pixel images. The evaluation was performed under a simulated Rician fading channel with the Rician factor set to $K_{R}=0.1$, corresponding to a severe multipath propagation environment with a weak line-of-sight (LoS) component. Testing was conducted over SNRs ranging from −12 dB to 15 dB with a step size of 3 dB.

To quantify the contribution of the SFRM, three variants of the STARS framework were implemented and evaluated:

1. STARS w/o SFRM: The STARS framework with the Semantic Feature Reweighting Module removed, where all extracted semantic features are transmitted without feature reweighting.
2. STARS with Static SFRM: The STARS framework equipped with a static semantic feature masking strategy, where a fixed proportion of semantic features is selected for transmission regardless of image content or channel conditions.
3. STARS (Proposed): The complete STARS framework employing the proposed adaptive Semantic Feature Reweighting Module, which dynamically adjusts feature importance according to image content.

**Figure 4:**
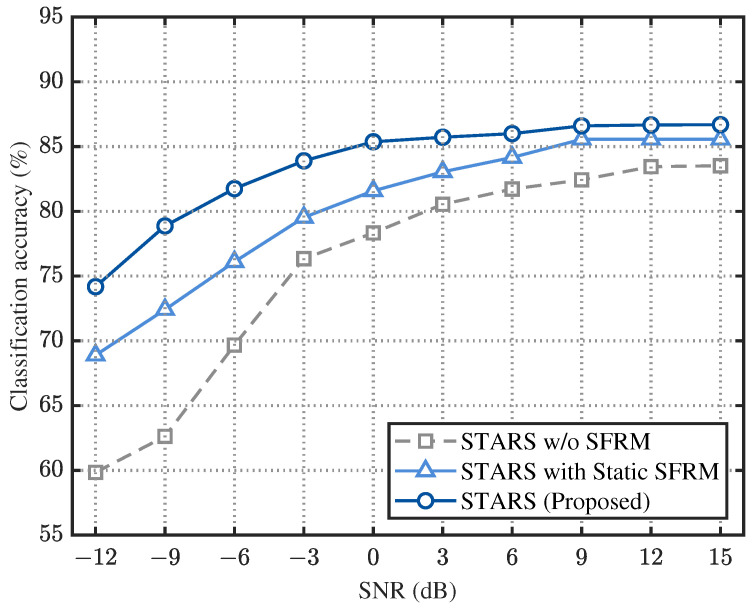
Quantitative performance comparison under Rician fading channels.

First, to evaluate the intrinsic semantic transmission reliability of the system, Figure 4 compares the auxiliary classification accuracy of different STARS variants under varying channel conditions. The complete STARS framework consistently achieves the best performance across the entire SNR range, with particularly significant gains observed in low-SNR scenarios.

At SNR=−12 dB, the STARS variant without SFRM achieves an Overall Accuracy of only 59.84% due to severe channel distortion. Introducing a static SFRM improves the OA to 68.89%, while the complete STARS framework further increases the OA to 74.18% through adaptive semantic feature reweighting. Compared with the variant without SFRM, the proposed framework achieves an absolute OA improvement of 14.34% under this challenging condition.

These results demonstrate the effectiveness of semantic feature reweighting in enhancing transmission robustness under adverse channel conditions. While static feature masking can partially suppress redundant information, the adaptive SFRM further improves performance by dynamically prioritizing task-relevant semantic features according to both image content and channel quality. Consequently, the impact of channel degradation on the transmitted semantic features is substantially mitigated, as reflected by the auxiliary classification metric.

As channel quality improves (SNR≥9 dB), the performance gap among different STARS variants gradually narrows. This trend suggests that semantic feature selection becomes less critical when transmission distortion is limited. Nevertheless, the complete STARS framework consistently maintains the highest auxiliary classification accuracy across all evaluated SNR conditions, demonstrating the robustness and general effectiveness of the proposed adaptive semantic feature reweighting strategy.

#### 5.3.2. Comparison with Conventional and Semantic Communication Methods

**Figure 5:**
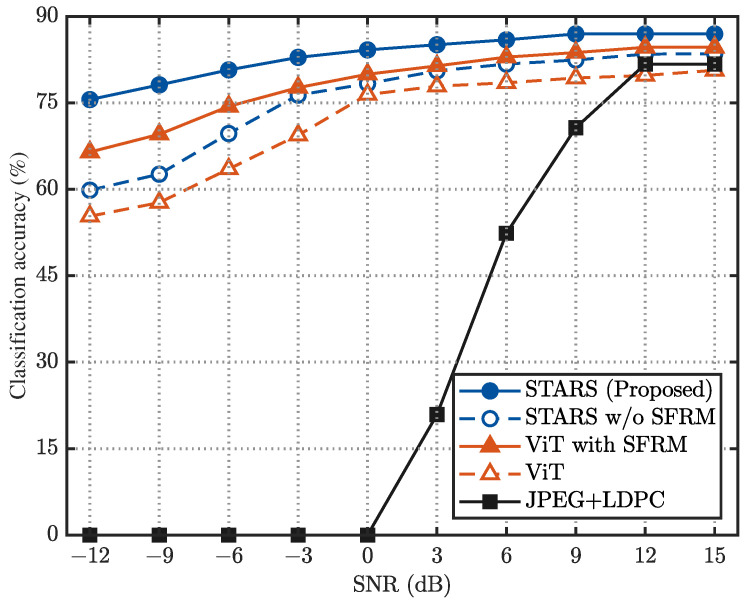
Performance comparison of conventional and semantic communication frameworks under varying channel conditions.

To further evaluate the contribution of the SFRM to the overall semantic communication framework, we compare the proposed STARS architecture with several representative baselines under different channel conditions. Since several recently proposed semantic communication approaches [20,21,22] are primarily designed for pixel-level image transmission and reconstruction rather than downstream object detection, they are not directly adopted as benchmarks here. Instead, Figure 5 illustrates the auxiliary classification accuracy across SNRs ranging from −9 dB to 15 dB.

- STARS (Proposed): The complete STARS framework integrating the Swin Transformer backbone and the proposed Semantic Feature Reweighting Module (SFRM).
- STARS w/o SFRM: The STARS framework with the SFRM removed.
- ViT with SFRM: Vision Transformer backbone equipped with the proposed SFRM.
- ViT: Semantic communication baseline based on the masked VQ-VAE scheme [26].
- JPEG + LDPC: Conventional transmit-then-process pipeline representing standard downstream vision frameworks operating under separate source-channel coding.

Figure 5 presents the performance comparison of conventional and semantic communication frameworks under varying channel conditions. Overall, all semantic communication approaches consistently outperform the conventional JPEG + LDPC baseline across the entire SNR range. It should be noted that this baseline represents the traditional transmit-then-process paradigm adopted by conventional recognition frameworks, where the original image must first be fully transmitted through a separate source-channel coding pipeline before downstream recognition can be performed.

As channel conditions deteriorate, the performance gap between conventional and semantic communication frameworks becomes increasingly pronounced. The JPEG + LDPC scheme exhibits a pronounced cliff effect under severe channel fading, resulting in a rapid collapse of auxiliary classification performance as transmission errors accumulate. In contrast, semantic communication methods exhibit substantially stronger robustness by directly preserving information relevant to downstream object detection.

Among all evaluated methods, the proposed STARS framework achieves the highest classification accuracy under all channel conditions. Even at SNR=−6 dB, STARS maintains an accuracy above 75%, significantly outperforming both the conventional communication baseline and the ViT-based semantic communication framework. These results demonstrate the effectiveness of the proposed architecture in preserving task-relevant semantic information under severe channel impairments.

A comparison between STARS and STARS w/o SFRM further verifies the contribution of the proposed Semantic Feature Reweighting Module. By adaptively emphasizing informative semantic features and suppressing redundant background content prior to transmission, the SFRM consistently improves auxiliary classification performance, particularly in low-SNR regimes where transmission resources are most constrained.

Furthermore, the comparison between the Swin-based and ViT-based semantic communication frameworks highlights the importance of hierarchical feature representation for remote sensing imagery. Benefiting from multi-scale feature extraction and shifted-window attention, the Swin Transformer backbone provides more discriminative semantic representations and achieves consistently superior performance across diverse channel conditions.

#### 5.3.3. Impact of Semantic Codebook Capacity on Transmission Efficiency and Accuracy

**Figure 6:**
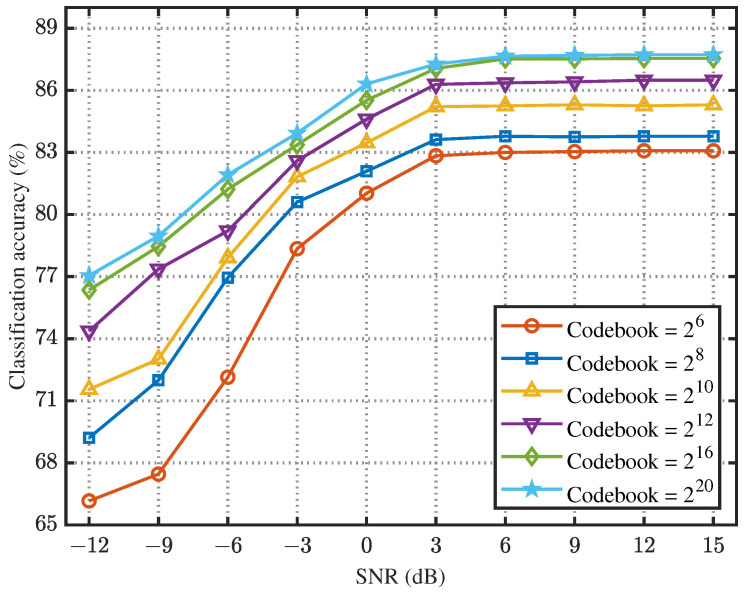
Target classification accuracy versus SNR for different semantic codebook sizes.

To investigate the impact of the discrete semantic codebook size on end-to-end performance and transmission overhead, comprehensive ablation studies were conducted on the DOTA dataset. In the STARS framework, the codebook capacity is determined by the quantization bit-width *B* (bits per digit, bpd), meaning the codebook contains $K=2^{B}$ basis vectors. The experiment compared the object detection accuracy of the system under varying SNRs for B∈{6,8,10,12,16,20}, and the results are illustrated in Figure 6.

As clearly observed from Figure 6, across the entire tested SNR range of −12 dB to +15 dB, the system’s detection accuracy initially exhibits a significant upward trend as the bit-width *B* increases. This performance leap is particularly pronounced in severe low-SNR environments. The underlying mechanism is that when *B* is small, the feature representation space of the codebook is severely constrained. Continuous, very-high-resolution semantic features are forced to project onto a highly limited number of basis vectors, inducing substantial irreversible quantization errors that strictly bottleneck the performance ceiling of downstream detection tasks.

However, the experimental results concurrently reveal that a larger codebook capacity is not unconditionally better; the system exhibits a pronounced phenomenon of diminishing marginal returns. When the quantization bit-width increases to B=16, the system’s detection accuracy approaches saturation. Furthermore, when *B* is further elevated to 20, the two performance curves nearly perfectly overlap, yielding no substantive accuracy gain. This phenomenon indicates that the codebook space at B=16 is sufficiently smooth and comprehensive to cover the effective semantic manifolds of complex aerial scenes. Blindly expanding the codebook beyond this point does not benefit object recognition. Instead, it drastically intensifies the computational complexity of onboard quantization matching and imposes an unnecessarily massive bit overhead on the downlink, which fundamentally contradicts the primary objective of semantic communication.

#### 5.3.4. Impact of Modulation Order on Transmission Robustness

**Figure 7:**
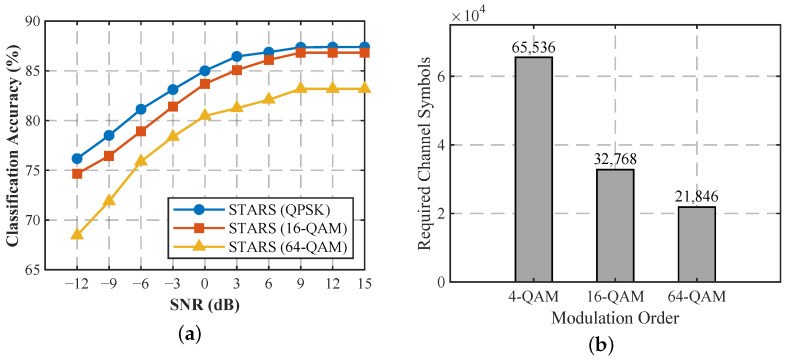
Impact of modulation order on transmission reliability and spectral efficiency in the proposed STARS framework. (**a**) Classification accuracy curves under QPSK, 16-QAM and 64-QAM across varying SNR conditions; (**b**) Comparison of transmission symbol count corresponding to different modulation orders.

To justify the selection of 16-QAM as the default modulation scheme, we evaluate the STARS framework under three varying modulation orders: 4-QAM, 16-QAM, and 64-QAM. All other physical-layer parameters, including the LDPC coding rate (1/2), VQ codebook capacity ($K=2^{16}$), and Rician fading conditions, remain strictly constant. As illustrated in Figure 7a, both transmission reliability and the subsequent downstream classification accuracy are highly sensitive to constellation density in low-SNR regimes. Unlike conventional communications where bit errors cause localized pixel perturbations, errors in the STARS framework corrupt discrete semantic indices. This leads to entirely incorrect codeword retrieval during VQ decoding, severely distorting the reconstructed feature manifold. Consequently, 64-QAM exhibits a noticeable performance degradation, as its dense constellation layout increases its vulnerability to channel noise. 4-QAM maximizes the Euclidean distance between symbols, maintaining robust accuracy even under severe fading conditions.

However, this robustness imposes a substantial penalty on spectral efficiency, as quantified in Figure 7b. Given the spatial dimensions of the semantic feature map (64×64), the system transmits 4096 discrete indices per image. With a $2^{16}$ codebook, each index corresponds to 16 bits, yielding a raw semantic payload of 65,536 bits. After rate-1/2 LDPC encoding, the total transmitted bitstream expands to 131,072 bits. Delivering this payload requires 65,536 channel symbols using 4-QAM. While 64-QAM compresses this overhead to approximately 21,846 symbols, its lack of noise resilience renders it impractical for volatile satellite links. 16-QAM strikes an optimal information-theoretic balance, requiring exactly 32,768 symbols while preserving near-optimal detection accuracy across a broad SNR range. This equilibrium between semantic fidelity and bandwidth compression fundamentally justifies its adoption within the proposed architecture.

#### 5.3.5. Benchmarking Results and Comparative Analysis

While the previous sections validated the intrinsic transmission robustness of the STARS framework via categorical accuracy, we now further evaluate its final downstream task utility using the instance-level object detection metrics. Having validated the efficacy of individual modules, we now evaluate the holistic performance of the STARS framework. Table 3 presents the quantitative comparison results against three mainstream object detection architectures (YOLOv8m, YOLOv10m, and Faster R-CNN) operating under the traditional transmit-then-detect paradigm, alongside a state-of-the-art semantic communication baseline, ViT-SemCom.

In terms of model complexity, the STARS framework demonstrates a favorable lightweight deployment characteristic. Benefiting from the hierarchical design of the Swin-T backbone and the compact semantic representation mechanism, the onboard semantic encoder of STARS contains only 19.7 M parameters, which is 23.9% fewer than YOLOv8m (25.9 M) and 29.9% fewer than the ViT-SemCom baseline (28.1 M). Although slightly larger than YOLOv10m, the proposed framework achieves a substantially better trade-off between model compactness and downstream semantic perception performance, making it highly suitable for resource-constrained satellite-borne platforms.

Regarding downstream task performance, STARS achieves consistently superior detection accuracy under the challenging Rician fading environment. The framework attains 69.3% mAP@0.5 and 47.1% mAP@0.5:0.95, outperforming comparison methods. Compared with Faster R-CNN under the traditional SSCC framework (58.3% mAP@0.5), STARS yields an absolute performance gain of 11.0%. More importantly, compared with the existing semantic communication baseline ViT-SemCom, STARS achieves improvements of 3.9% in mAP@0.5 and 4.0% in mAP@0.5:0.95. These results demonstrate that the proposed semantic-aware feature refinement and discrete representation mechanisms effectively enhance task-oriented semantic robustness while maintaining the fine-grained spatial structures required for high-fidelity bounding-box perception.

Furthermore, beyond the remarkable achievements in model compactness and detection accuracy, the STARS framework also demonstrates a highly competitive advantage in communication bandwidth efficiency. As detailed in Table 4, conventional deep JSCC approaches typically consume over 1 million channel symbols under a standard bandwidth ratio constraint of k/n=1/3. Similarly, conventional transmit-then-recognize pipelines, where mainstream intelligent perception frameworks rely on JPEG compression (Q50) followed by LDPC coding for image delivery, still necessitate approximately 460,000 transmitted symbols. In contrast, the proposed STARS architecture significantly reduces the communication burden, requiring merely 32,768 channel symbols and achieving an extremely compact actual bandwidth ratio of approximately 1/96.

This substantial reduction in transmission overhead is primarily enabled by the synergistic integration of the SFRM and the orthogonal discrete codebook. Unlike traditional pixel-level compression algorithms or standard deep JSCC schemes that transmit dense continuous features, STARS performs a fundamental paradigm shift toward discrete semantic representation. By aggressively filtering background clutter and transmitting only highly compact indices, this strategy ensures that the communication payload becomes proportional to the semantic density of the scene rather than its raw pixel resolution. Consequently, it provides an exceptionally bandwidth-efficient solution tailored for resource-constrained satellite-to-ground systems.

## 6. Conclusions

In this paper, we proposed STARS, a task-oriented semantic communication framework designed for efficient transmission and intelligent recognition of very-high-resolution satellite imagery in satellite-to-ground systems. Unlike conventional separate source-channel coding schemes that focus on pixel-level reconstruction, STARS establishes an end-to-end semantic transmission paradigm that directly optimizes downstream recognition performance. By integrating a Swin Transformer-based hierarchical semantic backbone, the proposed Semantic Feature Reweighting Module (SFRM), and a vector-quantized semantic coding mechanism, the framework effectively suppresses task-irrelevant background information and prioritizes the transmission of task-critical semantic features. Experimental results on the DOTA dataset under Rician fading channels demonstrate that STARS achieves strong robustness under adverse channel conditions. In particular, at an SNR of −12 dB, the dynamic STARS configuration achieved an accuracy of 74.18%, outperforming both the standard baseline and the static masking approach, confirming the effectiveness of channel-aware semantic feature allocation for robust satellite image transmission.

Future work will focus on further extending the proposed framework toward more practical cognitive space–ground networks. Specifically, we plan to investigate semantic communication for multi-modal remote sensing data, including SAR imagery and panchromatic bands, to improve cross-modal generalization capability. In addition, adaptive resource allocation strategies based on reinforcement learning will be explored to further enhance energy efficiency under strict onboard constraints. Finally, designing more efficient task-oriented semantic codebooks for multi-task downstream applications remains an important direction for future research.

## Figures and Tables

**Table 1:** Comparison of training convergence with and without the proposed SNR curriculum learning strategy.

Metric	Curriculum	No Curriculum
Best validation epoch	194	289
Early stopping epoch	244	339
Early stopping patience	50	50

**Table 2:** Architectural configuration and output sizes of the proposed STARS framework.

Part	Layers	Output Size
Encoder	Input Image	1024×1024×3
	Patch Embedding	256×256×96
	Stage 1 (2× Swin Blocks)	256×256×96
	Patch Merging 1	128×128×192
	Stage 2 (2× Swin Blocks)	128×128×192
	Patch Merging 2	64×64×384
	Stage 3 (6× Swin Blocks)	64×64×384
	SFRM Module (Semantic Reweighting)	64×64×384
	LayerNorm	64×64×384
Channel	Linear Bottleneck (Enc → Chan)	$4096\times C_{ch}$
	Vector Quantization (VQ, $K=2^{16}$)	$4096\times C_{ch}$
	PHY Layer (FEC + M-QAM + Fading)	Complex Symbols
	Receiver Processing (Demod + Decode)	$4096\times C_{ch}$
Decoder	Linear Projection (Chan → Dec)	64×64×96
	Dec Block 1 (Swin Block, W-MSA)	64×64×96
	Dec Block 2 (Swin Block, SW-MSA)	64×64×96
	Dec Block 3 (Swin Block, W-MSA)	64×64×96
	Dec Block 4 (Swin Block, SW-MSA)	64×64×96
	Global Average Pooling (MeanPool)	1×96
	Task-Specific Head (Linear)	$1\times N_{cls}$

**Table 3:** Performance comparison of different models on the DOTA-v1.0 dataset under a Rician channel at SNR = 6 dB.

Model	Backbone	Params (M)	mAP@0.5	mAP@0.5:0.95
YOLOv8m	CNN	25.9	63.5	40.8
YOLOv10m	CNN	16.5	62.1	39.6
Faster R-CNN	ResNet50	43.7	58.3	35.4
ViT-SemCom	ViT	28.1	65.4	43.1
STARS (Ours)	Swin	19.7	69.3	47.1

**Table 4:** Communication overhead comparison between STARS and conventional image transmission pipelines for downstream perception under a 1024×1024 RGB image transmission scenario.

Model	STARS (Ours)	Traditional Pipeline (JPEG+LDPC+Detector)	Deep JSCC
Transmitted symbols	32,768	∼460,000	1,048,576
Bandwidth ratio k/n	∼1/96	∼1/6.8	1/3

Note: *k* = transmitted complex symbols; *n* = 3,145,728 source symbols (pixels × color channels).

## Data Availability

Data will be made available on request.

## Nomenclature

Symbol	Description
I	Input VHR multispectral image
H,W	Image height and width
$B_{s}$	Training batch size
*N*	Number of patch tokens
*C*	Extracted semantic feature channels
${\hat{Y}}_{det}$	Downstream object detection predictions
X	Continuous semantic feature tensor
Q	Attention query matrix
K	Attention key matrix
V	Attention value matrix
$d_{q}$	Query/Key dimension
$B_{pos}$	Relative positional bias matrix in window attention
$Z_{hid}$	SFRM hidden semantic representation
m	Raw normalized importance score
M	Dynamic importance mask matrix
β	Mask lower bound threshold
$\hat{X}$	Refined feature tensor after semantic reweighting
g	Semantic importance logits generated by SFRM
$p_{aux}$	Auxiliary semantic classification logits
z	Pre-quantization continuous latent features
$d_{ch}$	Channel dimension for transmission
*B*	Quantization allocation bit-width
*K*	Discrete codebook capacity ($2^{B}$)
E	Shared discrete semantic codebook
$e_{k}$	The *k*-th discrete codeword
$k^{*}$	Matched 1D codeword indices
$z_{q}$	Quantized discrete semantic features
$\hat{z}$	Dequantized features at receiver
$\alpha_{ema}$	EMA smoothing decay rate
$V_{k}^{\left(t\right)}$	Codeword accumulated cluster volume
$v_{k}^{\left(t\right)}$	Codeword continuous feature sum
ϵ	Dead codeword reset threshold
$b_{str}$	Original binary information bitstream
c	LDPC-encoded error-resilient bitstream
s	Modulated complex transmitted symbols
S	Digital modulation constellation set
$N_{s}$	Total transmitted physical symbols
*P*	Average transmitted power constraint
*h*	Rician fading channel coefficient
$K_{R}$	Rician *K*-factor
n	Complex AWGN vector
$\sigma^{2}$	AWGN variance
y	Received physical signal vector
γ	Instantaneous Signal-to-Noise Ratio (SNR)
$L_{total}$	Total multi-task loss
η	Commitment cost coefficient

## Abbreviations

The following abbreviations are used in this manuscript:

STARS	Semantic Transmission Architecture for Remote Sensing
VHR	Very-High-Resolution
JSCC	Joint Source-Channel Coding
SSCC	Separate Source-Channel Coding
SFRM	Semantic Feature Reweighting Module
ViT	Vision Transformer
W-MSA	Window Multi-head Self-Attention
SW-MSA	Shifted Window Multi-head Self-Attention
VQ	Vector Quantization
EMA	Exponential Moving Average
STE	Straight-Through Estimator
LDPC	Low-Density Parity-Check
QAM	Quadrature Amplitude Modulation
AWGN	Additive White Gaussian Noise
SNR	Signal-to-Noise Ratio
LLR	Log-Likelihood Ratio
OBB	Oriented Bounding Box
mAP	Mean Average Precision
GF-2	Gaofen-2 Satellite
JL-1	Jilin-1 Satellite
